# Mitochondrial DNA mutation m.10680G > A is associated with Leber hereditary optic neuropathy in Chinese patients

**DOI:** 10.1186/1479-5876-10-43

**Published:** 2012-03-09

**Authors:** A-Mei Zhang, Xiaoyun Jia, Xiangming Guo, Qingjiong Zhang, Yong-Gang Yao

**Affiliations:** 1 Key Laboratory of Animal Models and Human Disease Mechanisms of Chinese Academy of Sciences & Yunnan Province, Kunming Institute of Zoology, Kunming, Yunnan 650223, China; 2 State Key Laboratory of Ophthalmology, Zhongshan Ophthalmic Center, Sun Yat-sen University, Guangzhou 510060, China

**Keywords:** LHON, mtDNA, m.10680G > A, Chinese, Rare primary mutation

## Abstract

**Background:**

Leber hereditary optic neuropathy (LHON) is a mitochondrial disorder with gender biased and incomplete penetrance. The majority of LHON patients are caused by one of the three primary mutations (m.3460G > A, m.11778G > A and m.14484T > C). Rare pathogenic mutations have been occasionally reported in LHON patients.

**Methods:**

We screened mutation m.10680G > A in the *MT-ND4L *gene in 774 Chinese patients with clinical features of LHON but lacked the three primary mutations by using allele specific PCR (AS-PCR). Patients with m.10680G > A were further determined entire mtDNA genome sequence.

**Results:**

The optimal AS-PCR could detect as low as 10% heteroplasmy of mutation m.10680G > A. Two patients (Le1263 and Le1330) were identified to harbor m.10680G > A. Analysis of the complete mtDNA sequences of the probands suggested that they belonged to haplogroups B4a1 and D6a1. There was no other potentially pathogenic mutation, except for a few private yet reported variants in the *MT-ND1 *and *MT-ND5 *genes, in the two lineages. A search in reported mtDNA genome data set (n = 9277; excluding Chinese LHON patients) identified no individual with m.10680G > A. Frequency of m.10680G > A in Chinese LHON patients analyzed in this study and our previous studies (3/784) was significantly higher than that of the general populations (0/9277) (*P *= 0.0005).

**Conclusion:**

Taken together, we speculated that m.10680G > A may be a rare pathogenic mutation for LHON in Chinese. This mutation should be included in future clinical diagnosis.

## Background

Leber hereditary optic neuropathy (LHON, MIM535000) is one of the most common mitochondrial disorders, which mainly leads to vision loss in young males [1-3]. Three primary mutations (m.3460G > A in the *MT-ND1 *gene, m.11778G > A in the *MT-ND4 *gene, and m.14484T > C in the *MT-ND6 *gene) accounted for the etiology of more than 95% LHON patients, whereas the remaining 5% cases was caused by rare mutations and/or unclear factors [1-3]. Up to now, there is a considerable long list for rare mutations for LHON (cf. http://www.mitomap.org). Most recently, m.3635G > A in the *MT-ND1 *gene was confirmed to be a rare primary mutation and had a multiple occurrence in Han Chinese and Russian LHON families [4-7]. Clinical expression of primary LHON mutation was affected by many factors including mtDNA background/haplogroups [8,9], nuclear genes [10-12] and environmental factors [2,13]. Though more and more LHON risk factors have been identified, there are abundant suspected LHON patients without any reported pathogenic mtDNA mutations [14,15].

In our recent study, we found mutation m.10680G > A in a suspected LHON family lacking any known primary mutation, and this family had a considerably high penetrance of disease (40%; [15]) compared with that of families with m.11778G > A (about 33.3%; [8]). Similarly, Yang *et al. *[16] reported one Chinese LHON family with both m.10680G > A and primary mutation m.14484T > C which expressed complete penetrance. These two studies suggested that m.10680G > A played an active role in LHON and might be a "suspected" pathogenic LHON mutation. In order to investigate the frequency of m.10680G > A in Chinese patients with clinical LHON features but without any known mutations, we screened this mutation in 774 suspected LHON patients by using the allele specific PCR (AS-PCR). Our analysis of the complete mtDNA genomes of the probands with mutation m.10680G > A suggested that this mutation should be regarded as a rare pathogenic mutation for LHON in Chinese.

## Materials and methods

### Patients

The patients were physically evaluated and collected at the Pediatric and Genetic Clinic of the Eye Hospital, Zhongshan Ophthalmic Center and/or other local clinical centers. All patients were subjected to acute or sub-acute vision loss and lacked the three known LHON primary mutations. We have sequenced the mtDNA control region sequence and classified these patients with suspected LHON into respective haplogroup and found no haplogroup was associated with suspected LHON [17]. Because we used up DNA samples for some patients during that study, only 774 out of 843 patients with suspected LHON [17] were analyzed here. All these 774 patients were confirmed to harbor none of the four LHON primary mutations (m.3460G > A, m.3635G > A, m.11778G > A, and m.14484T > C). Informed consents conforming to the tenets of the Declaration of Helsinki were obtained from each participant prior to the study. The institutional review boards of Zhongshan Ophthalmic Center and Kunming Institute of Zoology approved this study.

### Detection of mutation m.10680G > A

Mutation m.10680G > A was genotyped by using the AS-PCR in 774 suspected LHON patients. The primer pair for AS-PCR (L10680A: 5'-AGTCTTTGCCGCCTGCGATA-3'/H10972: 5'-TCAGGTAGTTAGTATTAGGAG-3') was designed according to the strategy described in Bi *et al. *[18]. Another primer pair L4887 (5'-TGACAAAAACTAGCCCCCATCT -3')/H5442 (5'-GCGATGAGTGTGGGGAGGAA-3') was used as the internal control for monitoring successful amplification during the AS-PCR. PCR was performed in a total volume of 20 μL containing 30 ng DNA, 10 mM Tris-HCl (pH 8.3), 1.5 mM MgCl_2_, 50 mM KCl, 0.5 units of TaKaRa rTaq, 175 μM of each dNTP, and 0.3 μM of each primer. The amplification condition for AS-PCR is composed of one denaturation cycle at 94°C for 3 min, 30 cycles of denaturation at 94°C for 20 s, annealing at 61°C for 20 s, and extension at 72°C for 30 s, and one final extension cycle at 72°C for 5 min. In order to evaluate the sensitivity of the AS-PCR in detecting the minimum level of heteroplasmic mutation m.10680G > A, five different concentrations of genomic DNA of patient Le1263 with m.10680G > A (1 ng, 2.5 ng, 5 ng, 10 ng, and 15 ng) was used for amplification. In addition, DNA samples from patient Le1263 and a healthy donor without m.10680G > A were mixed to achieve final proportions of mutant DNA of 5%, 10%, and 20%, and a total of 30 ng of mixed DNA was amplified. The sensitivity is defined to be the smallest percentage of mutant DNA that can be detected by the AS-PCR method. PCR products were separated on 1.5% agarose gel at 120 V for 30 min.

### Analysis of the entire mtDNA genome for patients with m.10680G > A

The entire mitochondrial genomes of probands with m.10680G > A were amplified and sequenced by using primers and methods described in our previous study [19]. Sequences were handled by the DNASTAR program (DNAS Inc, Madison, WI, USA). We classified each patient into accurate haplogroup relative to the updated East Asian mtDNA tree and PhyloTree [20-22]. The novelty of variants/mutations was defined according to the available guidelines described by Bandelt *et al. *[23]. We presented mtDNA sequence variations in probands sequenced in this study, together with two reported LHON mtDNAs with m.10680G > A (family Le1107 reported by Zou *et al. *[15] and LHON family reported by Yang *et al. *[16]) in a mtDNA tree. This phylogenetic approach has been demonstrated to be powerful to recognize private variants and haplogroup-specific variants in each lineage [23]. Evolutionary conservation analysis of m.10680G > A was performed by using the MitoTool (http://www.mitotool.org) [24].

To discern the frequency of m.10680G > A in reported mtDNAs across world, we collected 9277 complete (and/or incomplete) mtDNA sequences from PhyloTree (mtDNA Tree Build 12, 20 Jul 2011) [21] and the MitoTool data set [24] and excluded those data of Chinese LHON patients. The ten reported Chinese patients with suspected LHON in our recent study [15] were aggregated with the patients screened in this study. Two tailed Fisher Exact test was used to evaluate the difference of m.10680G > A frequency between Chinese patients with suspected LHON and the reported data. A *P *value less than 0.05 was considered as significant.

## Results

### Optimization of the AS-PCR for detecting m.10680G > A

**Figure 1:**
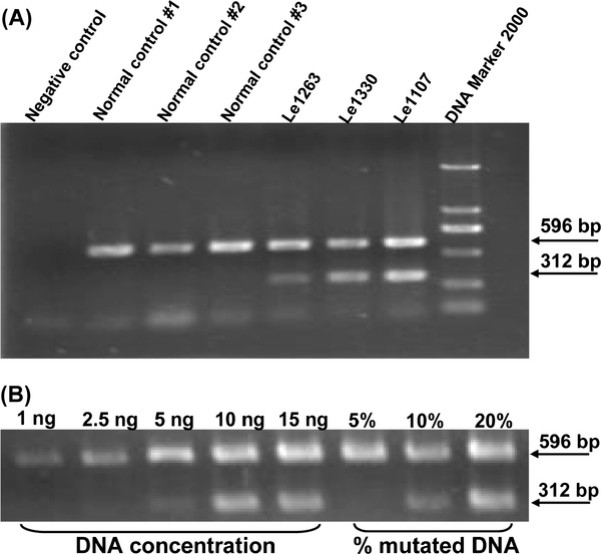
**Optimization of the allele-specific PCR method for detecting m.10680G > A**. The DNA template of proband Le1263 was diluted to have different concentrations with 1 ng, 2.5 ng, 5 ng, 10 ng, and 15 ng, respectively, to determine the smallest concentration of genomic DNA that could be detectable by the AS-PCR method. Normal controls were confirmed to lack mutation m.10680G > A. Patients Le1263, Le1330 (this study) and Le1107 in our previous study [15] harbored mutation m.10680G > A (A). Genomic DNA templates of patient Le1263 and normal control #1 were mixed to reach different proportions of mutant DNA. The percentage of mutated DNA stepwise from 5% to 20% (the mid-concentration is 10%), to determine the smallest percentage of mutant DNA (B). PCR products were separated on 1.5% agarose gel. The upper band (596 bp) is the internal control and the lower band (312 bp) indicates the presence of mutation m.10680G > A.

As demonstrated in Figure 1A, two bands could be amplified by using the allele-specific primer pair L10680A/H10972 (product length is 312 bp) and the internal control primer pair L4887/H5442 (product length is 596 bp) in the presence of mutation m.10680G > A. This AS-PCR approach had a relatively high sensitivity: 1) the specific band for m.10680G > A could be well recognized when 10 ng DNA was used, despite the fact that DNA template of 5 ng could also yield a weak target band; 2) we could detect m.10680G > A when it had a proportion of mutant DNA of 10% of total DNA template (Figure 1B).

### Clinical data and frequency of m.10680G > A in patients with suspected LHON

**Figure 2:**
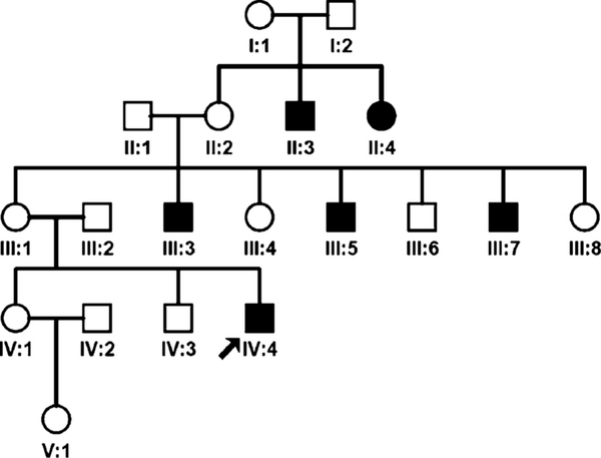
**Pedigree of LHON family Le1330 with m.10680G > A**. Filled symbols represent affected individuals with LHON. The proband that was analyzed for the complete mtDNA sequence was marked by an arrow.

We identified two patients (Le1263 and Le1330) with homoplasmic mutation m.10680G > A in 774 patients analyzed by the AS-PCR. Patient Le1263 is a 47 year-old man from Guangdong Province and has no self-reported family history of disease. He had felt an acute vision loss in both eyes for 33 days when he came to hospital. His best visual acuity is 0.2 for the right eye and 0.1 for the left eye at the time of his visit to our clinic. Fundus observation revealed mild pale of the optic disc in the right eye and mild edema of the optic disc with enlarged retinal vessels in the left eye. He had normal cornea, lens, and intraocular pressure (8 mmHg for the right eye and 11 mmHg for the left eye). Patient Le1330 is a 19 year-old man from Sichuan Province and has a family history of disease (Figure 2). He had acute vision loss for both eyes about 5 years ago. Ocular examination revealed pale temporal disc. Five other maternal relatives in his family had acute bilateral vision loss at around 30 years old (Figure 2), which is consistent with a pattern of maternal inheritance.

The overall frequency of m.10680G > A in Chinese patients with suspected LHON was considerably low (0.26% = 2/774), although this frequency was significantly higher than that of the complied mtDNA data set for world populations (0/9277) (*P *= 0.006). When we included ten suspected LHON patients with family history that were reported in our previous study [15], the total frequency increased to 0.38% (3/784) (compared to the complied mtDNA data, *P *= 0.0005). A web-based search and database search [23] showed that mutation m.10680G > A only occurred in Chinese LHON patients. The penetrance of LHON in family Le1330 was relatively high (6/14 = 42.9%), similar to the reported family Le1107 (8/20 = 40.0%) in our previous study [15]. All these results suggest that the rare mtDNA mutation m.10680G > A participates in the pathogenesis of LHON in Chinese.

### Multiple occurrence of m.10680G > A in different Chinese mtDNA lineages

**Figure 3:**
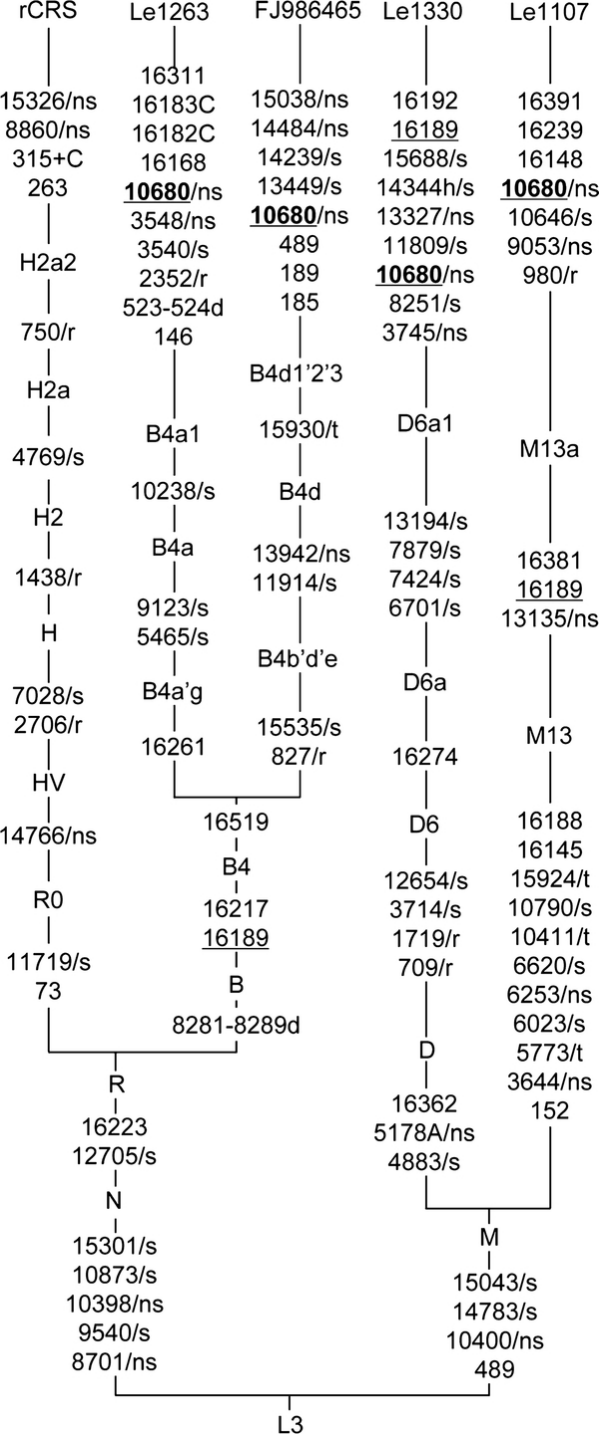
**Classification tree of four mtDNAs with m.10680G > A**. Two reported LHON mtDNAs (Le1107 [15] and FJ986465 [16]) were adopted from published sources. The revised Cambridge reference sequence (rCRS) [27] was included to show the phylogenetic relationship of the lineages and haplogroup-specific variants. The order of variants on each uninterrupted branch section is arbitrary. Haplogroup names were placed on each branch to show their hierarchical and nested positions within macrohaplogroups M and N. Recurrent variants were underlined. Deletion of nucleotide(s) was indicated by suffix "d". Length mutations of the C-tract in region 303-309 were omitted from the tree. Mutation m.10680G > A was marked in boldfaced. The synonymous and non-synonymous coding-region variants in these mtDNAs were further denoted by "/s" and "/ns", respectively. Variations in the ribosomal RNA genes and tRNA genes were denoted by "/r" and "/t", respectively.

Analysis for the complete mtDNA genomes of the two patients with m.10680G > A suggested that Le1263 and Le1330 belonged to haplogroups B4a1 and D6a1, respectively (Sequence could be retrieved from GenBank via accession numbers JN866824 and JN866825). Excluding these haplogroup-specific variants and mutation m.10680G > A, we found 11 private variants in Le1263 and 8 private variants in Le1330. Most of these private variants were synonymous or were located in the non-coding region. No "confirmed" or "suspected" LHON-associated mutation (which was listed in MITOMAP database: http://www.mitomap.org/bin/view.pl/MITOMAP/MutationsLHON) was found in these two patients (Figure 3 and Table 1). One non-synonymous variant in the *MT-ND1 *gene (m.3548T > C, p.I81T) and one variant in the *MT-RNR2 *gene (m.2352T > C) were found in patient Le1263, whereas patient Le1330 had two missense variants in the *MT-ND1 *gene (m.3745G > A, p.A147T) and the *MT-ND5 *gene (m.13327A > G, p.T331A). Except for variant m.2352T > C, the other three non-synonymous variants had a considerably high conservation index (CI) (> 0.8) (Table 1), suggesting these positions being evolutionarily conserved. All these private variants have been reported in the general populations, and variant m.3548T > C has been previously reported in patients with LHON [25] and diabetes [26].

**Table 1:** Private mtDNA variants in two Chinese probands with m.10680G>A

Sample	Nucleotide variant (Amino acid change)	Gene	**CI**^**a**^	**Population report**^**b**^	**Disease report**^**b**^
Le1263	m.3548T > C (p.I81T)	*MT-ND1*	0.907	Yes	Yes
	m.2352T > C	*MT- RNR2*	0.047	Yes	No
Le1330	m.13327A > G (p.T331A)	*MT-ND5*	0.837	Yes	No
	m.3745G > A (p.A147T)	*MT-ND1*	0.884	Yes	No

^a ^Conservation index (CI) was estimated by using 43 primate species according to the MitoTool [24].

^b ^The search was performed on Oct 31, 2011 following the same strategy described earlier [23] (e.g. both 'T3548C mtDNA' and 'm.3548T > C mtDNA' were queried).

## Discussion

Though over 95% LHON patients was affected by one of the three primary mutations, the etiological factor of the remaining 5% LHON patients was unclear [1-3]. Many sporadic cases without three primary mutations have been reported and mtDNA mutations identified in these lineages were considered as pathogenic, despite the fact that the exact pathogenicity remains to be proved by functional assays. The spectra of the LHON primary mutations showed remarkable difference between European patients and Chinese patients [8,9,28-30], we speculated that there might be some more pathogenic mtDNA mutations that were unique to Chinese LHON patients.

In this study, we designed an AS-PCR to detect mutation m.10680G > A in 774 Chinese patients with suspected LHON and found two patients harboring this mutation. Our approach had a reasonably good sensitivity in detecting minimum level of mutant DNA of 10% of total DNA template ≥ 10 ng). Compared to other approaches, such as sequencing, single-strand conformation polymorphism (SSCP), and restriction fragment length polymorphism (RFLP), the AS-PCR method is fast and cost-effective and can be of potential usage in the clinic for fast screening of mutation m.10680G > A in patients with suspected LHON. We did not identify any heteroplasmy of mutation m.10680G > A in the two patients with m.10680G > A in this study and patient Le1107 in our previous study [15] based on the limitation of detection sensitivity of our AS-PCR and direct sequencing approach. Though we did not design an allele-specific PCR (or a PCR-RFLP method) to detect the minimum level of wild-type allele in these patients to double check for heteroplasmy, the overall pattern was consistent with our previous observation for a very low frequency of heteroplasmic m.11778G > A (1/479 = 0.21%; Ref. [17]) and m.14484T > C (3/52 = 5.8%; Ref. [30]) in Han Chinese patients. As m.10680G > A creates a digestion site for enzyme *Tse*I, we could also use PCR-RFLP method to screen this mutation and measure the level of heteroplasmy. One limitation is that enzyme *Tse*I is quite expensive and this PCR-RFLP method is not cost-effective and time-consuming in clinic.

**Figure 4:**
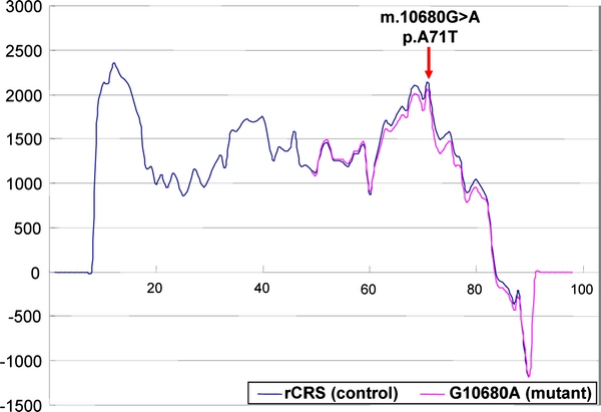
**Membrane-spanning region prediction plot produced by the TMpred program**. This program (http://www.ch.embnet.org/software/TMPRED_form.html) makes a prediction of membrane-spanning regions and their orientation. Mutation p.A71T (m.10680G > A) changed the structure of membrane-spanning region of the MT-ND4L protein.

The frequency of m.10680G > A in Chinese LHON patients was slightly lower than that of primary mutation m.3635G > A [7]. Although the conservation index (0.84) of p.A71T (m.10680G > A) was not as high as one would expect for a pathogenic mutation, it would affect the secondary or tertiary structure of the ND4L subunit (Figure 4). The alanine to threonine change at the 71^st ^amino acid position is next to E70, one of the two highly conserved glutamates in transmembrane helices of the ND4L protein, and those glutamates have been shown to be essential for enzyme activity [31]. Moreover, the two glutamates within transmembrane helices of nuoK/ND4L have been recently considered to be linked to the proton pumping function of complex I (NADH:ubiquinone oxidoreductase) in light of the recently revealed spatial structure of the membrane arm of complex I in *E. coli *[32,33]. Therefore, there indeed seem to be reasons for the pathogenicity of mutation m.10680G > A.

A web-based and database search showed that mutation m.10680G > A has been independently reported to affect LHON in two Chinese families [15,16]. These two reported patients belonged to haplogroups M13a [15] and B4d [16] (Figure 3). Therefore, it seemed that mutation m.10680G > A occurred only in Chinese LHON patients and had a multiple occurrence in different mtDNA background. In the Chinese family reported by Yang *et al. *[16], m.10680G > A coexisted with primary mutation m.14484T > C, and the presence of both mutations caused complete penetrance of LHON. In the two families with m.10680G > A reported in this study and our previous study [15], we found that the penetrance of LHON (> 40%) was even higher than those families with m.11778G > A (33.3%; Ref. [8]), m.14484T > C (31.9%; Ref. [30]) or m.3460G > A (25.6%; Ref. [29]). Patient Le1263 was self-reported as sporadic and his family was not considered to calculate the penetrance. Note that our estimation for disease penetrance for m.10680G > A was based on two families, which might lead to a bias.

Analysis for the entire mtDNA genomes of the two patients with m.10680G > A identified in this study showed that they harbored no novel or "confirmed" pathogenic mutation. However, variant m.3548T > C was found in patient Le1263, which is located in the mutational hotspot (*MT-ND1 *gene) for Chinese patients with LHON but lacking three known primary mutations [15] and was also reported in another LHON patient [25], whether m.3548T > C had a synergistic effect with m.10680G > A to influence disease expression in Le1263 was not clear, as we lacked necessary clinical information and this patient had no self-reported family history of disease. Similarly, two private variants were found in patient Le1330, which were located in the *MT-ND1 *and *MT-ND5 *genes (Table 1), further supported our previous claim that the *MT-ND1 *and *MT-ND5 *genes are mutational hotspots for Chinese families with clinical features of LHON but lacking the three primary mutations [15].

## Conclusion

In summary, we designed an AS-PCR method for rapid screening of m.10680G > A in a large cohort of Chinese patients with suspected LHON and identified two Chinese subjects with m.10680G > A. Analysis of the complete mtDNA sequences of the two probands and combining with information of two reported cases [15,16], we proposed that m.10680G > A is a rare pathogenic mutation in Chinese LHON population. Further functional assays, e.g. respiration measurements and NADH:ubichinon oxidoreductase activity assays in patient fibroblasts and eventually in newly constructed cybrid cell lines harbouring the mutation, should be carried out and more families with m.10680G > A should be included to validate our conclusion.

## Competing interests

The authors declare that they have no competing interests.

## Authors' contributions

AMZ and YGY designed the experiments and analyzed the data. AMZ performed the experiments. YGY, AMZ and QZ wrote the manuscript. XJ, XG and QZ provided the clinical data. All authors have read and approved the final manuscript.

## References

[B1] Man PYW Turnbull DM Chinnery PF Leber hereditary optic neuropathy J Med Genet 2002 39 162 169 10.1136/jmg.39.3.162 11897814 PMC1735056

[B2] Yu-Wai-Man P Griffiths PG Hudson G Chinnery PF Inherited mitochondrial optic neuropathies J Med Genet 2009 46 145 158 19001017 10.1136/jmg.2007.054270 PMC2643051

[B3] Carelli V Ross-Cisneros FN Sadun AA Mitochondrial dysfunction as a cause of optic neuropathies Prog Retin Eye Res 2004 23 53 89 10.1016/j.preteyeres.2003.10.003 14766317

[B4] Brown MD Zhadanov S Allen JC Hosseini S Newman NJ Atamonov VV Mikhailovskaya IE Sukernik RI Wallace DC Novel mtDNA mutations and oxidative phosphorylation dysfunction in Russian LHON families Hum Genet 2001 109 33 39 10.1007/s004390100538 11479733

[B5] Zhang A-M Zou Y Guo X Jia X Zhang Q Yao Y-G Mitochondrial DNA mutation m.3635 G > A may be associated with Leber hereditary optic neuropathy in Chinese Biochem Biophys Res Commun 2009 386 392 395 10.1016/j.bbrc.2009.06.051 19527690

[B6] Yang J Zhu Y Tong Y Chen L Liu L Zhang Z Wang X Huang D Qiu W Zhuang S Ma X Confirmation of the mitochondrial ND1 gene mutation G3635A as a primary LHON mutation Biochem Biophys Res Commun 2009 386 50 54 10.1016/j.bbrc.2009.05.127 19497304

[B7] Jia X Li S Wang P Guo X Zhang Q mtDNA m.3635 G > A may be classified as a common primary mutation for Leber hereditary optic neuropathy in the Chinese population Biochem Biophys Res Commun 2010 403 237 241 10.1016/j.bbrc.2010.11.017 21074518

[B8] Ji Y Zhang A-M Jia X Zhang Y-P Xiao X Li S Guo X Bandelt H-J Zhang Q Yao Y-G Mitochondrial DNA haplogroups M7b1'2 and M8a affect clinical expression of leber hereditary optic neuropathy in Chinese families with the m.11778 G > A mutation Am J Hum Genet 2008 83 760 768 10.1016/j.ajhg.2008.11.002 19026397 PMC2668067

[B9] Hudson G Carelli V Spruijt L Gerards M Mowbray C Achilli A Pyle A Elson J Howell N La Morgia C Clinical expression of Leber hereditary optic neuropathy is affected by the mitochondrial DNA-haplogroup background Am J Hum Genet 2007 81 228 233 10.1086/519394 17668373 PMC1950812

[B10] Phasukkijwatana N Kunhapan B Stankovich J Chuenkongkaew WL Thomson R Thornton T Bahlo M Mushiroda T Nakamura Y Mahasirimongkol S Genome-wide linkage scan and association study of PARL to the expression of LHON families in Thailand Hum Genet 2010 128 39 49 10.1007/s00439-010-0821-8 20407791

[B11] Abu-Amero KK Jaber M Hellani A Bosley TM Genome-wide expression profile of LHON patients with the 11778 mutation Br J Ophthalmol 2010 94 256 259 10.1136/bjo.2009.165571 19726426

[B12] Zhang A-M Jia X Zhang Q Yao Y-G No association between the SNPs (rs3749446 and rs1402000) in the PARL gene and LHON in Chinese patients with m.11778 G > A Hum Genet 2010 128 465 468 10.1007/s00439-010-0875-7 20711738

[B13] Kirkman MA Yu-Wai-Man P Korsten A Leonhardt M Dimitriadis K De Coo IF Klopstock T Chinnery PF Gene-environment interactions in Leber hereditary optic neuropathy Brain 2009 132 2317 2326 10.1093/brain/awp158 19525327 PMC2732267

[B14] Ferré M Bonneau D Milea D Chevrollier A Verny C Dollfus H Ayuso C Defoort S Vignal C Zanlonghi X Molecular screening of 980 cases of suspected hereditary optic neuropathy with a report on 77 novel OPA1 mutations Hum Mutat 2009 30 E692 705 10.1002/humu.21025 19319978

[B15] Zou Y Jia X Zhang A-M Wang W-Z Li S Guo X Kong Q-P Zhang Q Yao Y-G The MT-ND1 and MT-ND5 genes are mutational hotspots for Chinese families with clinical features of LHON but lacking the three primary mutations Biochem Biophys Res Commun 2010 399 179 185 10.1016/j.bbrc.2010.07.051 20643099

[B16] Yang J Zhu Y Tong Y Zhang Z Chen L Chen S Cao Z Liu C Xu J Ma X The novel G10680A mutation is associated with complete penetrance of the LHON/T14484C family Mitochondrion 2009 9 273 278 10.1016/j.mito.2009.04.003 19394449

[B17] Zhang A-M Jia X Bi R Salas A Li S Xiao X Wang P Guo X Kong Q-P Zhang Q Yao Y-G Mitochondrial DNA haplogroup background affects LHON, but not suspected LHON, in Chinese patients PLoS One 2011 6 e27750 10.1371/journal.pone.0027750 22110754 PMC3216987

[B18] Bi R Zhang A-M Yu D Chen D Yao Y-G Screening the three LHON primary mutations in the general Chinese population by using an optimized multiplex allele-specific PCR Clin Chim Acta 2010 411 1671 1674 10.1016/j.cca.2010.06.026 20599858

[B19] Wang H-W Jia X Ji Y Kong Q-P Zhang Q Yao Y-G Zhang Y-P Strikingly different penetrance of LHON in two Chinese families with primary mutation G11778A is independent of mtDNA haplogroup background and secondary mutation G13708A Mutat Res 2008 643 48 53 10.1016/j.mrfmmm.2008.06.004 18619472

[B20] Kong Q-P Sun C Wang H-W Zhao M Wang W-Z Zhong L Hao X-D Pan H Wang S-Y Cheng Y-T Large-scale mtDNA screening reveals a surprising matrilineal complexity in East Asia and its implications to the peopling of the region Mol Biol Evol 2011 28 513 522 10.1093/molbev/msq219 20713468

[B21] van Oven M Kayser M Updated comprehensive phylogenetic tree of global human mitochondrial DNA variation Hum Mutat 2009 30 E386 394 10.1002/humu.20921 18853457

[B22] Kong Q-P Bandelt H-J Sun C Yao Y-G Salas A Achilli A Wang C-Y Zhong L Zhu C-L Wu S-F Updating the East Asian mtDNA phylogeny: a prerequisite for the identification of pathogenic mutations Hum Mol Genet 2006 15 2076 2086 10.1093/hmg/ddl130 16714301

[B23] Bandelt H-J Salas A Taylor RW Yao Y-G Exaggerated status of "novel" and "pathogenic" mtDNA sequence variants due to inadequate database searches Hum Mutat 2009 30 191 196 10.1002/humu.20846 18800376

[B24] Fan L Yao Y-G MitoTool: a web server for the analysis and retrieval of human mitochondrial DNA sequence variations Mitochondrion 2011 11 351 356 10.1016/j.mito.2010.09.013 20933105

[B25] Yen MY Wang AG Chang WL Hsu WM Liu JH Wei YH Leber's hereditary optic neuropathy-the spectrum of mitochondrial DNA mutations in Chinese patients Jpn J Ophthalmol 2002 46 45 51 10.1016/S0021-5155(01)00460-9 11853713

[B26] Crispim D Estivalet AA Roisenberg I Gross JL Canani LH Prevalence of 15 mitochondrial DNA mutations among type 2 diabetic patients with or without clinical characteristics of maternally inherited diabetes and deafness Arq Bras Endocrinol Metabol 2008 52 1228 1235 10.1590/S0004-27302008000800005 19169474

[B27] Andrews RM Kubacka I Chinnery PF Lightowlers RN Turnbull DM Howell N Reanalysis and revision of the Cambridge reference sequence for human mitochondrial DNA Nat Genet 1999 23 147 10.1038/13779 10508508

[B28] Jia X Li S Xiao X Guo X Zhang Q Molecular epidemiology of mtDNA mutations in 903 Chinese families suspected with Leber hereditary optic neuropathy J Hum Genet 2006 51 851 856 10.1007/s10038-006-0032-2 16972023

[B29] Yu D Jia X Zhang A-M Guo X Zhang Y-P Zhang Q Yao Y-G Molecular characterization of six Chinese families with m.3460 G > A and Leber hereditary optic neuropathy Neurogenetics 2010 11 349 356 10.1007/s10048-010-0236-7 20232220

[B30] Yu D Jia X Zhang A-M Li S Zou Y Zhang Q Yao Y-G Mitochondrial DNA sequence variation and haplogroup distribution in Chinese patients with LHON and m.14484 T > C PLoS One 2010 5 e13426 10.1371/journal.pone.0013426 20976138 PMC2956641

[B31] Kervinen M Patsi J Finel M Hassinen IE A pair of membrane-embedded acidic residues in the NuoK subunit of Escherichia coli NDH-1, a counterpart of the ND4L subunit of the mitochondrial complex I, are required for high ubiquinone reductase activity Biochemistry 2004 43 773 781 10.1021/bi0355903 14730982

[B32] Efremov RG Sazanov LA Structure of the membrane domain of respiratory complex I Nature 2011 476 414 420 10.1038/nature10330 21822288

[B33] Efremov RG Sazanov LA Respiratory complex I: 'steam engine' of the cell? Curr Opin Struct Biol 2011 21 532 540 10.1016/j.sbi.2011.07.002 21831629

